# Obstructive Hydrocephalus and Cardiomyopathy Secondary to Disseminated Protothecosis in a Boxer Dog

**DOI:** 10.1155/2024/1402828

**Published:** 2024-08-12

**Authors:** Anna Tauro, John Macri, Chris Gaudette, Christopher L. Mariani, Bonnie Brenseke

**Affiliations:** ^1^ College of Veterinary Medicine North Carolina State University, Raleigh, North Carolina, USA; ^2^ Pathology Department Campbell University, Buies Creek, North Carolina, USA

**Keywords:** brain, culture, cytology, dog, formalin-fixed paraffin-embedded tissues, heart, histology, MRI, panfungal PCR, *Prototheca*

## Abstract

Canine protothecosis is a rare disease caused by saprophytic unicellular achlorophyllous aerobic algae that are ubiquitous in the environment. We report a novel case of neurological and cardiological manifestations associated with disseminated protothecosis. An adult spayed female Boxer dog was presented with a 1-week history of anorexia, progressive central vestibular signs, and a Grade III/VI systolic heart murmur. Magnetic resonance (MR) imaging revealed obstructive hydrocephalus at the level of the mesencephalic aqueduct, while echocardiography and elevated troponin levels suggested an infiltrative cardiomyopathy. No obvious cause was identified. Cerebrospinal fluid (CSF) collection was not performed due to associated procedural risks. Despite receiving symptomatic treatment and maintaining stability for 3 weeks, the dog eventually suffered cardiorespiratory arrest. Postmortem examination revealed disseminated protothecosis, predominantly affecting the heart and brain. We recommend that in cases where the cause of obstructive hydrocephalus is unclear, especially when CSF collection is not feasible, a comprehensive diagnostic method should be implemented. This includes meticulous investigations to identify infected tissues, followed by sampling and performing cytology/histology and culture tests to confirm the presence of the algal organism. Early diagnosis may allow early treatment, although long-term prognosis remains largely unfavorable due to the absence of effective treatments.

## 1. Introduction

Protothecosis, an infectious disease of humans and animals, is caused by *Prototheca* spp., which are saprophytic unicellular achlorophyllous aerobic algae that are ubiquitous in the environment and distributed nearly worldwide [1–3].

The species of *Prototheca* recognized as pathogenic to dogs include *P. wickerhamii* and *P. zopfii* [4]. *P. zopfii* is the most common cause of protothecal mastitis in cows and disseminated protothecosis in dogs [4]. Recent revisions in *Prototheca* spp. taxonomy have led to the reclassification of *P. zopfii* Genotypes 1 and 2 as *P. ciferrii* and *P. bovis*, respectively [5, 6], with *P. bovis* being notably virulent and responsible for the disseminated forms in dogs [7]. Canine protothecosis appears to be overrepresented in young adult, spayed female Collies and Boxers [3, 4, 8]. Often initial signs are referable to the gastrointestinal tract, such as chronic intermittent diarrhea and hematochezia, suggesting an oral (ingestion) route of infection followed by secondary hematogenous or lymphatic dissemination [3, 4, 9–12]. The grave prognosis of this disease is believed to be due to delayed diagnosis; therefore, early suspicion and detection of the disease might enable early treatment, preferably before the dissemination of infection. [8, 9]

The abundance of algal organisms in the affected tissue makes cytological or histological examination highly sensitive diagnostic tools [7]. As more than half of affected dogs shed algae into their urine, routine investigation should include urine culture, urine sedimentation for cytology, and deep rectal scraping for cytological examination [4, 7]. Despite the ability to culture *Prototheca* spp. on standard media [7], the lack of standardized susceptibility testing makes the clinical significance of the results uncertain [7, 13]. Currently, no universally accepted treatment regimen exists in human or veterinary medicine, although combinations of antifungal and antibacterial agents, especially amphotericin B with azoles, have shown some efficacy [4, 10, 14]. The duration of therapy is still not determined, and because prolonged treatment is often necessary, many animals may experience adverse reactions to the medications used [2].

Cases of canine protothecosis presenting with neurological manifestations but no clinical evidence of disseminated infection are sparsely reported, and these cases lack evidence of cerebrospinal fluid (CSF) flow obstruction or hydrocephalus [10, 15–18]. Two case reports documented disseminated canine protothecosis with CSF outflow obstruction and consequent dilation of the third and fourth ventricles [3] or fourth ventricle [2] on magnetic resonance (MR) images. However, these findings were observed alongside multifocal T2-weighted hyperintense brain parenchymal lesions due to protothecal granulomatous inflammation [2, 3, 17]. Another case report described moderate third and lateral ventriculomegaly on brain computed tomography (CT), though it remained uncertain whether these findings were normal for this specific patient or indicative of obstructive hydrocephalus [19].

In this report, we describe a case of a 6-year-old spayed female Boxer dog exhibiting progressive neurological signs associated with obstructive hydrocephalus, in the absence of discernible lesions on MR images. This study is aimed at detailing both MRI and histopathological findings in a dog with both neurological and cardiological manifestations associated with disseminated protothecosis, a presentation not previously documented.

## 2. Case Presentation

### 2.1. History

A 6-year-old, 18.5 kg, spayed female Boxer dog was presented with a 1-week history of anorexia and progressive paraparesis. The dog was up to date on vaccinations and received routine prophylactic treatment for heartworms, fleas, and ticks. Previously, the dog had a history of chronic intermittent vomiting, diarrhea, and reduced appetite for 2–3 years, which resolved with symptomatic therapy, including metronidazole, maropitant, and dietary change. Notably, no gastrointestinal signs were reported for over a year leading up to the current presentation.

The dog was born and has lived entirely in North Carolina (USA), with no reported history of travel outside the state.

Initial investigations performed by the referring veterinarian, including blood work (hematology, serum biochemistry), and SNAP 4Dx Plus Test for heartworm disease, Lyme disease, ehrlichiosis, and anaplasmosis, along with fecal flotation, yielded no significant abnormalities.

### 2.2. Investigations, Treatments, and Outcome

Upon physical examination, the dog had a body condition score of 3 out of 9, nasal hyperkeratosis, and a Grade III/VI systolic heart murmur. The dog appeared quiet but responsive. Neurological assessment revealed paraparesis, with mild delays in postural responses noted in both pelvic limbs and the left thoracic limb. The remainder of the neurological examination yielded normal findings.

Hematology and serum biochemistry revealed mild hyperglobulinemia (4.9 g/dL, reference range 2.5–4.5) along with elevated levels of amylase (1627 U/L, reference range 500–1500) and lipase (1944 U/L, reference range 200–1800). The dog was admitted 4 days later for advanced imaging. By this time, neurological signs had worsened, with the dog exhibiting intermittent left head turn, vestibular ataxia with a tendency to fall to either side, positional rotary nystagmus with a fast phase to the right (clockwise), and reduced left pupillary light reflex. Mentation and postural reactions remained unchanged. Suspicions arose regarding a multifocal infectious, inflammatory, or neoplastic process affecting the brain (forebrain and brainstem).

Repeat hematology indicated mild neutrophilia (9.235 × 10^3^/UL, reference range 2.841–9.112) and lymphopenia (0.351 × 10^3^/UL, reference range 0.594–3.305). Thoracic radiographs showed no abnormalities.

Echocardiography revealed moderate left ventricular (LV) concentric hypertrophy, dynamic LV outflow tract obstruction (DLVOTO), and systolic anterior motion of the anterior mitral valve leaflet, suggestive of possible mitral valve dysplasia. Increased vagal tone, evident as pronounced respiratory sinus arrhythmia, was also observed. During the atropine challenge test, the dog exhibited a normal rhythm transitioning to sinus tachycardia; however, DLVOTO worsened with the increased heart rate. No treatment was deemed necessary. Elevated troponin levels (1.12 ng/mL, reference range < 1.0) suggested an infiltrative cardiomyopathy.

**Figure 1:**
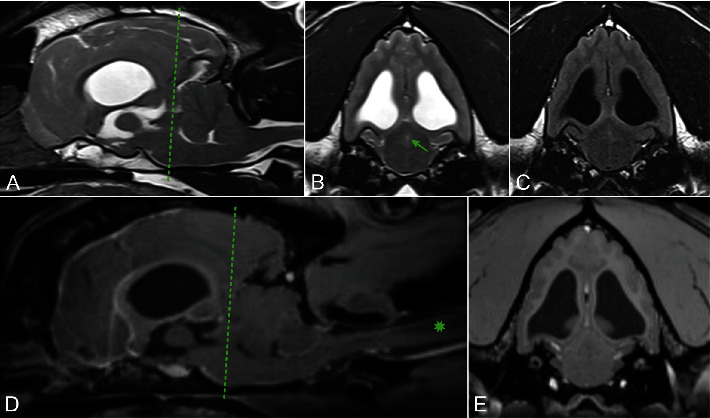
Magnetic resonance images. (A) T2-weighted midsagittal plane. (B) T2-weighted transverse plane and (C) T2-weighted FLAIR transverse plane at the level of the aqueduct. (D) T1-weighted midsagittal fat-saturated postcontrast plane. (E) T1-weighted transverse fat-saturated postcontrast plane. The MRI reveals CSF flow obstruction at the level of the aqueduct (green arrow) with no discernible cause. Additionally, transtentorial herniation with flattening of the rostral aspect of the cerebellum and foramen magnum herniation with associated cervical syringomyelia (green star) were observed, likely attributed to increased intracranial pressure. CSF = cerebrospinal fluid; FLAIR = fluid-attenuated inversion recovery; MRI = magnetic resonance imaging.

Brain MRI was performed using 3.0 Tesla unit (Siemens Medical Solutions USA, Inc., Malvern, Pennsylvania), and multiple sequences were acquired, including T2-weighted, proton density (PD)-weighted images, T2-weighted-fluid-attenuated inversion recovery (T2-FLAIR), diffusion-weighted imaging (DWI) and apparent diffusion coefficient (ADC) maps, and susceptibility weighted imaging (SWI). Multiplanar reconstruction of T1-weighted, fat-saturated images pre and postintravenous contrast mediums was obtained. The findings revealed obstructive hydrocephalus at the level of the mesencephalic aqueduct (Figure 1).

This was associated with moderate dilation of the lateral and third ventricles, as well as the suprapineal recess of the third ventricle. Additionally, transtentorial herniation with flattening of the rostral aspect of the cerebellum and foramen magnum herniation and associated cervical syringomyelia were noted, likely secondary to increased intracranial pressure. No obstructive cause was identified. Occipital malformation and absence of the septum pellucidum were considered congenital and treated as incidental findings.

CSF collection was not performed due to the suspected elevated intracranial pressure and associated procedural risks. *Neospora caninum* and *Toxoplasma gondii* (IgM and IgG) serological testing yielded negative results. A fungal serology panel returned negative findings for *Cryptococcus* antigen (microagglutination method) and *Histoplasma*, *Coccidioides*, and *Blastomyces* antibodies (agar gel immunodiffusion). However, *Aspergillus* antibodies (agar gel immunodiffusion) were detected at a dilution of 1:4. This finding was considered to be clinically insignificant.

PCR testing yielded negative results for *Anaplasma* spp., *Anaplasma platys*, *Anaplasma phagocytophilum*, *Babesia* spp., *Phylum Apicomplexa*, *Bordetella* spp., *Ehrlichia* spp., hemotropic *Mycoplasma* spp., and *Rickettsia* spp. Immunofluorescence testing showed seronegativity (< 1:32) for *Rickettsia rickettsia*, *Bartonella vinsonii*, *Bartonella henslae*, *Bartonella koehlerae*, *Ehrlichia canis*, *Babesia canis*, and *Babesia gibsoni*. Furthermore, SNAP4Dx Plus test for *Dirofilaria immitis* antigen, antibody to *Anaplasma phagocytophilum*, antibody to *Anaplasma platys*, antibody to *Borrelia burgdorferi*, antibody to *Ehrlichia canis*, and antibody to *Ehrlichia ewingii* returned negative results.

Following the MRI and while awaiting the infectious disease test results, the dog was discharged with a tapering course of prednisone (Prednisone; Mylan Pharmaceuticals Inc., Morgantown, West Virginia) at an anti-inflammatory dose (0.8 mg/kg PO SID for 3 days, then 0.5 mg/kg SID), along with omeprazole (Omeprazole, Lek Pharmaceuticals D.D., Ljubljana, Slovenia) (1 mg/kg PO SID). Following a stable period of 3 weeks, the dog's neurological status rapidly deteriorated, with the dog becoming obtunded. Regrettably, upon readmission to the hospital, the dog experienced a cardiorespiratory arrest, and the cardiopulmonary resuscitation was unsuccessful. Due to this sudden deterioration, we were unable to perform additional diagnostic tests.

### 2.3. Postmortem Examination

Postmortem examination was performed. Grossly, the dog was very thin with significant fat and muscle loss and had severe nasal hyperkeratosis and multifocal small hemorrhages (petechiae and ecchymoses) along the medial thighs. The heart was discolored white tan, enlarged, and thickened (weight 214 g; 1.16% of body weight; normal is < 1%).

The LV free wall, right ventricular free wall (RV), and interventricular septum measure 1.3 cm, 0.3 cm, and 1.8 cm, respectively (LV:RV ratio of 4.3 : 1; reference range: 2-4 : 1) [20]. The liver lobes were small (hypoplastic) except the right medial lobe, which was large (hyperplastic, suggestive of compensation) [20]. There was mild mesenteric lymph node enlargement. The brain had moderate dilation of the lateral ventricles, and a 4 mm section of the cerebellum was compressed between the brainstem and the overlying bone (cerebellar herniation). Fractured ribs and subjacent pulmonary hemorrhage were attributed to the resuscitation efforts.

**Figure 2:**
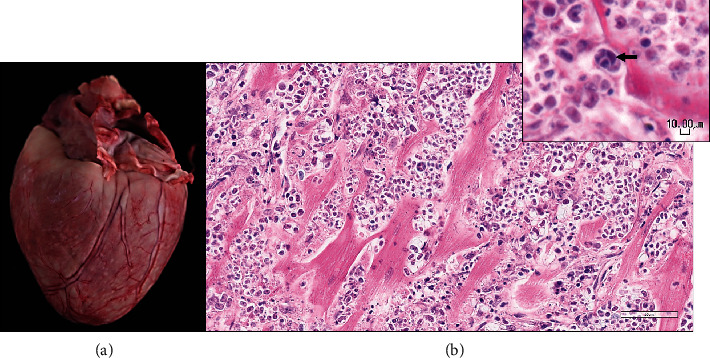
Postmortem examination, heart. (a) Grossly, the heart is enlarged and discolored white tan. (b) Histologically, the heart is infiltrated and largely replaced by algae and associated necrosis and inflammation. Endosporulation is evident; endospores are occasionally arranged in a radial tripartite configuration (“Mercedes-Benz” endospores; arrow) consistent with *Prototheca*; hematoxylin and eosin (H&E), 400x; inset-H&E, 600x.

**Figure 3:**
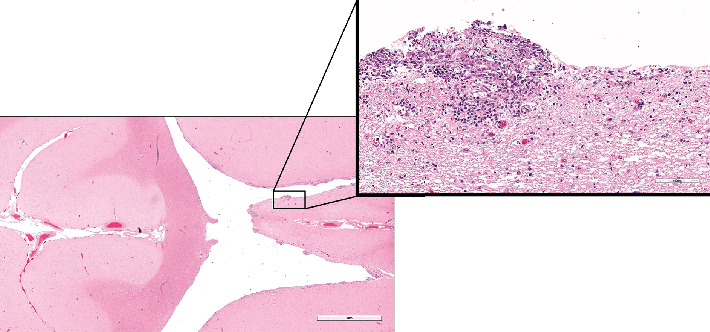
Brain histology. Algae and associated inflammation line and bulge into ventricles; H&E, 20x and 200x (inset).

**Figure 4:**
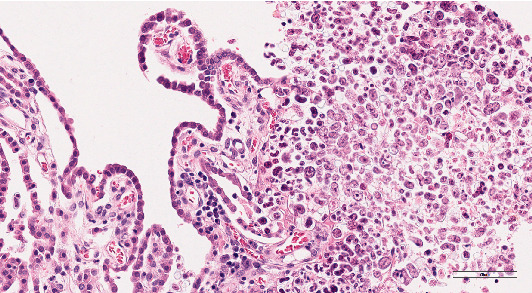
Brain histology. Algae infiltrate and expand the choroid plexus; H&E, 200x.

**Figure 5:**
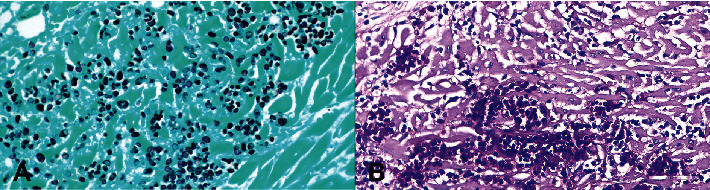
Heart histology. (A) Organisms stain positively with Grocott's methenamine silver and (B) periodic acid–Schiff with diastase, compatible with *Prototheca* infection.

Histological examination was performed on the heart, lung, mediastinal lymph node, liver, spleen, ileum, cecum, colon, colonic lymph node, kidney, bone marrow, skin (from medial thigh), nose, and brain. Findings revealed disseminated protothecosis, with algae detected in the heart, brain, ileum, and skin. Algal organisms were most numerous in the heart and brain (Figures 2, 3, and 4), and these organs had an associated inflammatory infiltrate composed of lymphocytes, plasma cells, and macrophages. Organisms were 5–15 *μ*m in diameter with a round to oval basophilic nucleus and a relatively thick clear wall. Some were empty and collapsed with only the cell wall remaining. Many had undergone endosporulation (Figure 2), in which the sporangia had wedge-shaped, radially arranged, angular endospores (daughter cells) divided by septations. Endospores were occasionally arranged in a radial tripartite configuration (“Mercedes-Benz” endospores) consistent with algae, specifically *Prototheca*. Moreover, organisms stained with Grocott's methenamine silver (GMS), periodic acid–Schiff (PAS), and periodic acid-Schiff with diastase (PAS-D) (Figure 5). In the brain, algal organisms were associated with proliferative lymphoplasmacytic and granulomatous inflammation of the ventricles and choroid plexus.

The organisms and resultant inflammation bulged into the ventricular system, likely causing the obstructive hydrocephalus. The heart was largely effaced by algae and associated inflammation and necrosis. The rapid deterioration and death of this animal were attributed to the severity of disease in the vital organs of the brain and heart.

The histomorphology of the organisms strongly suggested a *Prototheca* infection. As panfungal PCR can sometimes identify *Prototheca* spp., attempts were made to detect this alga via panfungal PCR and sequencing on formalin-fixed paraffin-embedded (FFPE) tissues. Given the abundance of algae observed in the heart, we focused our testing on heart tissue. Unfortunately, despite multiple attempts, good-quality DNA was unable to be amplified, leading to the inability to more definitively identify the specific *Prototheca* species.

*Chlorella*, a green alga, bears a striking resemblance to *Prototheca* in its microscopic appearance [7]. Although no green discoloration of the tissues was seen grossly in this case and although disseminated chlorellosis in the dog is exceedingly rare [21], we performed special stains to more definitively rule out *Chlorella*. Periodic acid-Schiff staining detects polysaccharides and starch granules, with diastase acting as an enzyme that digests these granules. Whereas *Chlorella* has PAS-positive diastase-sensitive cytoplasmic starch granules, our test confirmed that the organisms in this case were PAS-positive with diastase-resistant endospores, consistent with *Prototheca* spp. (Figure 5).

## 3. Discussion

Diagnosing canine protothecosis can be challenging, particularly when systemic manifestations such as gastrointestinal or ocular signs are absent. In our case, identifying the exact route of infection remains elusive. However, based on the history and postmortem examination, gastrointestinal or integumentary routes appear most probable.

The most reliable method for diagnosing *Prototheca* spp. involves cytology or histopathological evaluation of infected tissues, complemented by routine culture [4]. In cases where the central nervous system is involved, CSF analysis is crucial. This test may detect *Prototheca* organism [2, 12] or show either eosinophilic [4, 12, 15, 16] or lymphocytic [2, 3, 19] pleocytosis, assisting in diagnosis. Additionally, the aerobic culture of CSF could yield positive results [2, 3]. CSF collection was not conducted in our case due to perceived risks in the face of a suspected increase in intracranial pressure. This limitation hindered the diagnosis of the disease.

In this case, the histomorphology, including the special stain findings, is consistent with *Prototheca* infection. Real-time PCR assays have been developed for the detection and differentiation of *Prototheca* species, but these are not commercially available for diagnostic purposes in animals [7]. An attempt at speciation was made via the performance of panfungal PCR and sequencing on FFPE heart tissue; however, good-quality DNA was unable to be amplified. Additionally, the possible presence of *Chlorella*, a green alga resembling *Prototheca*, was ruled out based on the negative result of PAS staining with diastase.

Previous studies have established *Prototheca*'s tropism for well-vascularized tissues like the myocardium and central nervous system [4, 9]. The severity of the neurological disorder and cardiomyopathy in our case suggests that they could have both contributed to the sudden deterioration and death of this dog. Although canine protothecosis has been linked to ventriculomegaly in the context of multifocal brain lesions [2, 3, 19], the combination of obstructive hydrocephalus without an identifiable obstructive lesion and severe cardiomyopathy seen in our case has not been previously reported.

Given the high sensitivity of cytological examination and culture test for diagnosing algal infections [7], alternative diagnostic strategies should be employed when CSF collection is not feasible. We propose that in cases where the cause of obstructive hydrocephalus is unclear, meticulous investigations to identify infected tissues, followed by sampling and performing cytology/histology and culture tests, should be considered to confirm the presence of the algal organisms, even in the absence of systemic signs. In our case, the detection of *Prototheca* in the ileum suggests that a more comprehensive gastrointestinal work-up, including cytology/histology and culture of multiple intestinal sampling, might have facilitated an earlier diagnosis. The skin lesions observed on the medial thighs during postmortem examination were not noted or missed during physical examination, highlighting the importance of a complete integumentary examination in these cases. Furthermore, the identification of a newly diagnosed heart murmur should alert clinicians to the possible presence of a systemic disorder.

*Prototheca* species are opportunistic pathogens capable of causing infections in both immunocompetent and immunosuppressed patients. However, more severe and disseminated infections are more likely to occur in immunocompromised individuals [7, 13]. It is noteworthy that Boxer dogs seem to be disproportionately affected by canine protothecosis, possibly secondary to an underlying genetic immunodeficiency [4, 6, 7, 15, 16, 22]. We believe that the dog in this case report was likely immunocompromised and showed evidence of a congenital (potentially genetic) disease, as indicated by a liver anomaly.

There is no effective treatment for canine protothecosis. The lack of diagnosis in our case prevented us from initiating specific therapy. Early detection and intervention, ideally before the onset of the disseminated form, are recommended. However, despite such efforts, the prognosis typically ranges from poor to grave. Combination therapy with multiple azoles has been reported to be potentially more beneficial [4]. Given the disseminated form of the disease in dogs, drugs capable of crossing the blood-brain barrier are preferred. Based on limited reported cases, amphotericin B (especially the liposomal formulation, administered intravenously or intrathecally), fluconazole, and posaconazole appear to be the most effective [2, 3]. Additionally, amphotericin B enemas have been explored as a novel approach for cases with gastrointestinal involvement [8]. However, it is important to note the potential for significant side effects, such as nephrotoxicity with amphotericin B and hepatotoxicity with azole antifungal agents, necessitating vigilant monitoring. Additionally, the prolonged duration of treatment required to manage the disease, often spanning months to years, poses a considerable financial burden for owners [4]. Anti-inflammatory doses of corticosteroid therapy have been used in canine protothecosis to reduce CSF production and the inflammatory response to infectious disease [2]. However, the long-term use and immunosuppressive doses of corticosteroids in immunocompromised human patients are known to significantly increase host susceptibility to *Prototheca* spp. [7, 23]. Therefore, it remains uncertain whether the use of steroids at anti-inflammatory doses in this case report had any benefits or detrimental effects.

## 4. Conclusions

Obstructive hydrocephalus and cardiomyopathy can be associated with the disseminated form of canine protothecosis.

If the severity of the neurological status precludes CSF collection, we recommend that in cases where the cause of obstructive hydrocephalus is unclear, meticulous investigations to identify infected tissues, followed by sampling and performing cytology/histology and culture tests, should be considered to confirm the presence of the algal organisms.

Early diagnosis and treatment may be beneficial; however, to date, the long-term prognosis for canine protothecosis remains poor.

## Data Availability

Data sharing is not applicable to this article as no datasets were generated or analyzed during the current study.
